# Assessment of a County-Wide, Pan-First Responder Take-Home Naloxone Program in Denver, Colorado

**DOI:** 10.1016/j.acepjo.2026.100357

**Published:** 2026-03-13

**Authors:** Joshua Jacoves, Alexandra Mannerings, Donald Stader, Mario Andres Camacho, James Neuenschwander, Ansley Mason, Katelynn Petrasic, Scott G. Weiner

**Affiliations:** 1 The Naloxone Project, Denver, Colorado, USA; 2 Epifluence, Denver, Colorado, USA; 3 Department of Emergency Medicine, Denver Health Medical Center, University of Colorado School of Medicine, Denver, Colorado, USA; 4 Department of Emergency Medicine, The Ohio State University College of Medicine, Columbus, Ohio, USA; 5 Department of Emergency Medicine, Brigham and Women’s Hospital, Boston, Massachusetts, USA

## Abstract

**Objectives:**

To evaluate the feasibility and geospatial distribution of a county-wide pan-first responder leave-behind naloxone program implemented across emergency medical services (EMS), police, fire, and a community response mobile crisis unit.

**Methods:**

We conducted a retrospective analysis of leave-behind naloxone kits distributed in Denver, Colorado, from April 26, 2024, through April 25, 2025. Trained first responders (*n* ∼2850) recorded each kit distribution via an online form capturing location, agency, recipient category, and transport disposition. Descriptive statistics were used to summarize kit distributions by agency and transport outcomes. A hotspot analysis mapped clusters of distributions and assessed proximity to transit stops and other public facilities.

**Results:**

Over the 12-month period, there were 1020 interactions during which responders distributed 2438 naloxone kits. Paramedics accounted for 1407 kits (57.7%), the mobile crisis unit for 642 (26.3%), police for 336 (13.8%), and fire for 53 (2.2%). In many interactions, the kit recipient declined transport, particularly in the mobile crisis unit and EMS cohorts. Geospatial analysis identified 24 hotspots, of which 23 were within feasible walking distance of bus or light-rail stops. These findings suggest that leave-behind naloxone distributions cluster around transportation hubs in Denver.

**Conclusion:**

A pan-first-responder leave-behind naloxone program is feasible at the county level, with most kits distributed by paramedics and a mobile crisis unit program. Hotspot analysis highlighted transit-adjacent clusters, informing potential sites for prestationed naloxone. Cross-agency collaboration combined with geospatial targeting may rapidly saturate high-risk settings with naloxone.

**The Bottom Line**

A county-wide pan-first responder leave-behind naloxone program in Denver demonstrated that distributing naloxone through multiple first-response agencies is feasible at scale. Over a 12-month period, responders distributed 2,438 naloxone kits during 1,020 encounters, with most kits provided by paramedics (57.7%) and the mobile crisis response program (26.3%). Geospatial analysis showed that 23 of 24 distribution hotspots occurred near bus or light-rail stops, suggesting that transit hubs may be strategic locations for pre-positioning naloxone for bystander use. Overall, multi-agency collaboration combined with geospatial targeting may represent a scalable strategy to rapidly increase naloxone access in communities at highest risk for opioid overdose.

## 1 Introduction

### 1.1 Background

Naloxone has emerged as a primary modality for reversing opioid overdoses and preventing overdose deaths, serving as a cornerstone of US drug policy.^1^ Communities that had greater dispensation of nasal naloxone experienced fewer overdose-related deaths compared with those that did not have overdose education and nasal naloxone distribution programs.^2^ As a result, there is a large initiative to expand access to naloxone, including the US Food and Drug Administration’s approval of an over-the-counter formulation of nasal naloxone spray,^3^ and roll-out of vending machines and public-access boxes^4^^,^^5^ that can equip bystanders and people who use drugs with naloxone in case they encounter an overdosing person.

Another potentially impactful initiative is “leave-behind” naloxone, in which first responder agencies provide naloxone to patients, bystanders, and individuals on scene.^6^ There is evidence that 5% to 7% of people who survive an overdose die within the year following the index nonfatal overdose event,^7^, ^8^, ^9^ indicating that providing those at risk with naloxone before the overdose occurs may be protective. Additionally, these individuals may encounter others nearby experiencing an opioid overdose and may be crucial to reverse an overdose within their social circle, including family, friends, and other community members. People who use drugs express support for leave-behind naloxone.^10^

### 1.2 Importance

In 2023, the City and County of Denver, Colorado, began the process of implementing a program of leave-behind naloxone through 4 categories of first responders (“pan-first responder”): police, fire, emergency medical services (EMS), and a social support program called Support Team Assisted Response (STAR, a community response mobile crisis unit). Success of the program would be demonstrated by the feasibility of a high-efficiency naloxone distribution system that provides a large number of naloxone kits through a range of first-response agencies, beyond just EMS, to be implemented in other jurisdictions. Because first responders increasingly encounter individuals experiencing overdose or other drug-related complications,^11^ this targeted intervention is hypothesized to be an effective, scalable, and reproducible method to distribute naloxone and address the opioid public health emergency.

### 1.3 Goals of This Investigation

This study aimed to describe the experience of the first 12 months of a county-wide, leave-behind naloxone distribution program. The primary aim was to determine the number of kits provided on scene. Secondary aims included determination of naloxone administration by a bystander prior to first responder arrival, the proportion of naloxone provided by fire and police as opposed to medical first responders, if the call was related to overdose, and if the individual was transported to the hospital. We also aimed to geocode the areas where naloxone was distributed to determine optimal locations to prestation naloxone for bystander use.

## 2 Methods

### 2.1 Study Design and Setting

This was a retrospective analysis of reported leave-behind naloxone episodes by first responder agencies in the City and County of Denver, Colorado. The program was funded through an Opioid Abatement grant from the Denver Opioid Abatement Council. Naloxone 4 mg nasal spray was purchased at the public interest price utilizing grant funds and was distributed via The Naloxone Project, a Colorado-based nonprofit organization, to participating agencies. The Naloxone Project developed the training program through consultation with first responders, emergency physicians, and EMS physicians. First responders were trained during their shifts and were paid accordingly by their respective agencies. The Mass General Brigham Institutional Review Board approved the study (Protocol #2025P000753).

### 2.2 Selection of Participants

The study worked to capture all of Denver’s transporting first-response agencies. To achieve this, program administrators partnered with the Denver Health Paramedic Division, the primary transporting ambulance agency), the Denver Fire Department, the Denver Police Department (DPD), and Denver STAR. Denver STAR deploys teams of behavioral health clinicians, paramedics, and emergency medical technicians to address low-risk 911 calls related to behavioral health, substance use, and homelessness, providing immediate and sometimes extended crisis intervention and connections to relevant services. Interfacility Transport Agencies were excluded from the intervention because they do not respond directly to 911 calls.

Denver Health Paramedic Division is the provider of EMS in the City and County of Denver. At the time of the intervention, this agency had a fleet of 36 ambulances and approximately 300 responders (Basic Life Support and Advanced Life Support), responding to >140,000 calls annually. Additionally, toward the end of the study period, Denver Health Paramedic Division implemented community engagement bicycle shifts, where EMS providers canvassed the city, giving out naloxone kits to those they determined to be in need. These distributions were included in the overall number attributed to Denver Health Paramedic Division. STAR operates 8 response vans with approximately 50 staff. DPD operates out of 6 police stations across the city, with an additional station at the Denver International Airport. The department has approximately 1500 staff, with each officer carrying 1 or 2 leave-behind naloxone kits in their respective service vehicle (eg, car, bicycle, motorcycle, and quick-response unit). Denver Fire Department operates 39 fire stations across the city, with approximately 1000 employed firefighters. The department also staffs 5 substations at Denver International Airport. After a case review of all Denver International Airport calls at these 5 substations, the choice was made not to stock kits at the airport fire stations due to anticipated low numbers of applicable cases and DPHD’s ability to leave-behind naloxone at this location. Denver has an approximate population of 729,000 individuals, with an increasing number of unhoused individuals (estimated 6539 in 2024 and 7327 in 2025).

As all categories of first-response agencies in the city participated and multiple agencies often responded to the same call, training emphasized a hierarchy to determine which agency should distribute kits. If Denver Health Paramedic Division medics were on scene, they would be the primary kit giver, followed by STAR, then DPD, and Denver Fire Department. This order was chosen as Denver Health Paramedic Division and STAR often had the most contact with the kit recipient and could instruct them on naloxone administration and provide resources.

### 2.3 Interventions

The intervention began after securing partnership agreements with all agencies. Trainers with expertise in emergency medicine, addiction medicine, harm reduction, and prehospital care provided training for frontline responders (approximately *n* = 2850) (DPD ∼1500, Denver Fire Department ∼1000, Denver Health Paramedic Division ∼300, STAR ∼50). Trainings were held during March 2024 and consisted of in-person training at stations. Prehospital responders unable to attend in-person training completed asynchronous virtual training. Training included information on the logistics of a leave-behind naloxone interaction (eg, eligible patient and nonpatient populations, how to communicate with participants, and how to use the kits) and the concept of therapeutic reconciliation. Therapeutic reconciliation is a novel technique that encourages first responders to explore the origins of their internalized stigma, frequently linked to challenging interactions with patients with substance use disorder, and to address it through harm reduction strategies like leave-behind naloxone. The training also included instructions on how to record each interaction with leave-behind naloxone. The intervention was launched in each agency once at least 90% of its frontline responders had completed either in-person or online training. The first distribution of naloxone as part of this initiative occurred by Denver Health Paramedic Division on April 26, 2024.

Frontline providers were directed to distribute leave-behind naloxone to patients who, based upon their best clinical judgment, were either currently experiencing substance or opioid use disorder or to patients who were deemed at increased risk for opioid overdose, given relevant medical or environmental factors. Providers were also directed to distribute kits to nonpatients who were in a position to assist those at risk of overdose, including bystanders, friends, family members, and peers who were also present at the scene.

### 2.4 Measurements

Data were collected from April 26, 2024, to April 25, 2025. Participating responders were instructed to fill out a short online data form (Supplementary Appendix 1) at the location and time of naloxone distribution. A unique QR code leading to the data form was created for each agency and placed either on their identification badges or as a decal on the compartment housing leave-behind naloxone kits. The form required input of the following variables: (1) address of distribution; (2) if the individual at risk of overdose received an administered dose of naloxone prior to responder arrival; (3) the number of naloxone kits distributed to the at-risk individual; (4) the number of naloxone kits distributed to individuals in a position to assist those at risk for an opioid overdose (eg, bystander, family member or friend on scene); and (5) transport decision. An additional optional question asked for details about the experience with distributing naloxone for internal quality improvement purposes. The address could be entered manually or populated automatically using cellular phone triangulation.

### 2.5 Outcomes

The key outcome measure was the number of naloxone kits distributed, both in total and stratified by type of responder agency. Distribution was also stratified by the following factors: if naloxone was administered by a bystander prior to first responder arrival, the proportion of naloxone provided by fire and police as opposed to medical first responders, if the call was related to overdose, if naloxone was distributed to nonpatient individuals on scene, and if the individual was transported to the hospital. The address of distribution was collected for 3 purposes: (1) to identify areas where overdose was most likely to occur so that agencies could focus attention to those areas; (2) to identify agencies that may not be distributing naloxone when appropriate (eg, if 1 agency consistently distributed kits in 1 specific hotspot but another did not, it would prompt further investigation); and (3) to identify locations for prestationing of naloxone (eg, in vending machines) in future interventions.

### 2.6 Analysis

Data collection elements were analyzed using descriptive statistics. Geospatial analysis was used to examine hotspots of distribution points via a Getis-Ord Gi∗ statistic analysis.^12^^,^^13^ These hotspot clusters were then overlaid on relevant points of interest (bus routes and stops, light rail routes and stops, shelters, and public health entities) that could serve as potential areas for prestationing naloxone. To determine the parameters for hotspots, we utilized the rough geography of Denver, assuming an average block size of 100 m^2^ and then used a 3-block radius to approximate hotspots. Hotspots that clearly fit these parameters are shown in red, and hotspots with atypical geometry, ie, those that did not form a circle, or that were close to our predetermined parameters, are shown in yellow.

## 3 Results

### 3.1 Main Results

Over the 12 months of the study period, there were 1020 interactions during which the 4 agencies distributed a total of 2438 leave-behind naloxone kits: Denver Health Paramedic Division *n* = 1407 (57.7%), STAR *n* = 642 (26.3%), DPD *n* = 336 (13.8%), and Denver Fire Department *n* = 53 (*n* = 2.2%). The Table describes the number of kits distributed and outcomes stratified by agency.

**Table:** Description of number of naloxone kits and outcomes stratified by agency.

Agency name	No. of interactions	No. of kits to patients	No. of kits to nonpatients	Total no. of kits dispensed	Percentage of interactions where a kit was distributed and the call type was “overdose”^a^	Percentage of patients who refused transport for all call types	Percentage of overdose calls where naloxone was administered prior to EMS arrival
Denver Health Paramedics	713	780	627	1407	67%	67%	57%
Denver STAR	263	287	355	642	20%	95%	38%
Denver Police	23	149	187	336	34%	50%	25%
Denver Fire	21	22	31	53	71%	37%	53%
Total	1020	1238	1200	2438	54%	73%	55%

a An overdose interaction was defined as an incident where a first responder noted overdose as the primary or secondary impression as via our data collection form.

**Figure 1:**
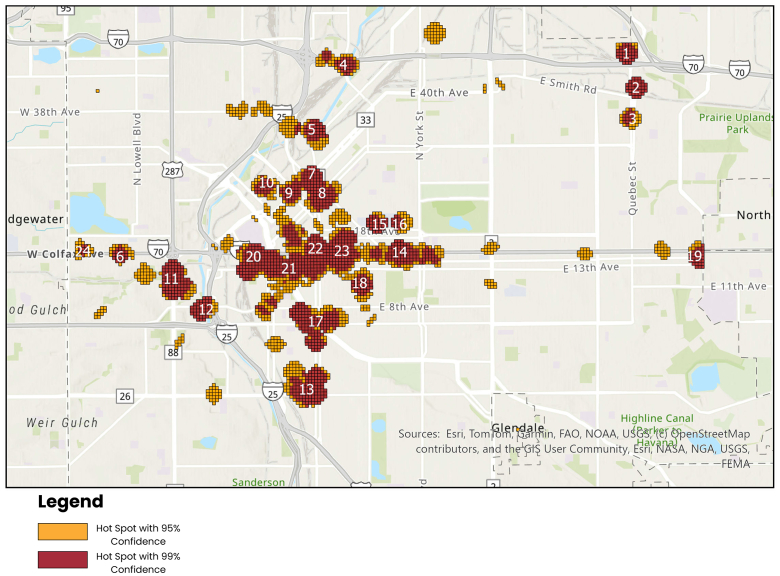
Hotspots where leave-behind naloxone was most likely to occur.

**Figure 2:**
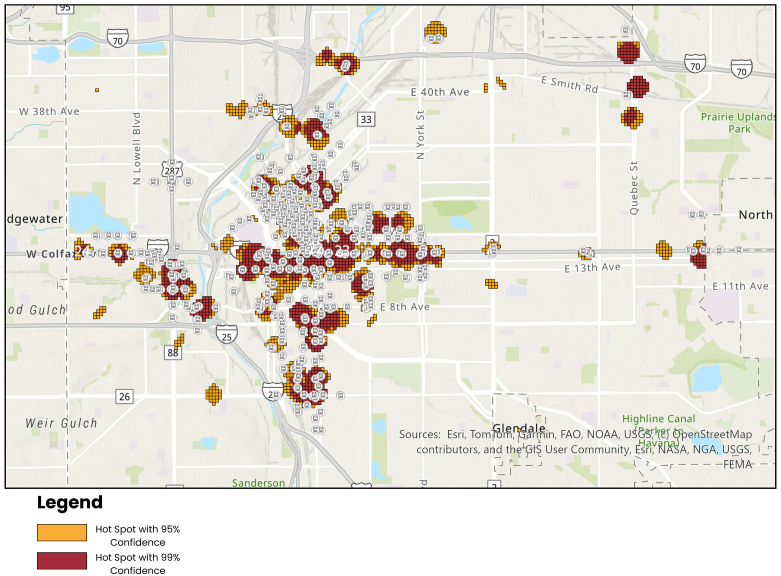
Hotspots where leave-behind naloxone was most likely to occur, with transit stations located within that hotspot superimposed.

Through our Gi∗ statistic analysis, we identified 24 hotspots meeting our predefined criteria, with each representing at least 3 kits distributed in a 600-meter geographic area (Figure 1). Out of these 24 hotspots, 23 contain at least 1 Denver Regional Transportation District bus stop or light rail station. The 16th hotspot is a recently established tiny-home community for previously unhoused individuals in the city. Figure 2 demonstrates hotspots where leave-behind naloxone was most likely to occur, with transit stations located within that hotspot superimposed.

## 4 Limitations

The program was conducted in a single county with a robust network of transit and community response mobile crisis units, limiting generalizability to rural or resource-limited settings. Although most of the City and County of Denver was covered, some areas were not due to some towns being unincorporated or their agencies not participating in the program. Because distribution events were self-reported via a QR code-link online form, some may have been missed, and we could not determine the total number of eligible encounters or how many kits were distributed without completion of the online data form; moreover, some kits were given proactively to individuals deemed at risk rather than in response to an overdose, including by EMS bicycle units. Relatedly, we only had access to data from the online form and not call logs or medical records, which prevented cross-checking against the medical records. This limitation was also structural, as naloxone could be dispensed to nonpatients who would not have a medical record. Finally, we could not assess whether dispensed kits were ultimately used or if they influenced subsequent patient outcomes. More research will be needed to determine patient-level outcomes after receiving a kit, the cost-effectiveness of the program, strategies to improve documentation, and whether the distribution of kits was equitable across neighborhoods and racial or ethnic groups. In addition to the aforementioned limitations are the inherent problems of retrospective data analysis, which include missing or inconsistent data, inability to prove causation, and lack of control over data collection. Potential missing data could have introduced bias in both the descriptive and spatial analyses.

## 5 Discussion

In this county-wide evaluation of a pan-first responder naloxone leave-behind program, 2438 kits were distributed across 1020 distinct interactions over 1 year. Most kits were given by Denver Health Paramedic Division (paramedics) or the STAR mobile crisis response program, whereas police and fire distributed fewer kits, presumably because Denver Health Paramedic Division is frequently also dispatched for medical calls. It was by design that paramedics and STAR dispensed most kits, as they have the most patient contact and medical knowledge.

The finding that 23 of 24 distribution hotspots were near bus or light-rail stops suggests that people receiving kits may congregate around transportation hubs, highlighting the importance of integrating geospatial considerations into naloxone deployment programs, as has been done for public automated external defibrillator deployments. Further research is needed to determine how far someone is willing to walk or run to get naloxone instead of staying with an overdosing victim while awaiting EMS providers. It is also unclear whether bus stops are a viable location, given that naloxone would be subject to temperature changes or the elements, and logistics such as inventory control and restocking may be challenging.

Our experience demonstrates that a citywide leave-behind naloxone program spanning EMS, law enforcement, fire, and social service units is feasible and can potentially saturate high-risk settings. Although a formal evaluation of free-text comments on the electronic form was not conducted, general themes of gratitude for the program, as well as reports of kits being distributed to bystanders who had recently used a kit and needed a replacement, were common, suggesting perceived value by first responders. Moreover, our study illustrates that coordinated training, data collection, and protocol adoption between first responder agencies in a large US city are feasible and can result in significant, actionable data to inform further public health efforts to address the overdose crisis. These results build on prior leave-behind programs in urban and suburban settings. San Francisco’s Project First Responder Increased Education and Naloxone Distribution provided 1200 kits to EMS providers in 2019.^14^ The Howard County Department of Fire and Rescue Services distributed 120 naloxone kits from June 2018 to June 2019.^15^ This evaluation found that when kits were left with a family member on scene or a friend, the patient was more likely to be connected to treatment and recovery resources. Leave-behind naloxone is one of the many creative solutions that was implemented in Ohio as part of the HEALing Community Study.^16^

For a successful program, it is important to garner the support of key stakeholders and enact relevant laws and regulations as necessary. A survey of the Michigan leave-behind naloxone program found that most EMS field providers and administrators were supportive of the program, but some were concerned about creating unintended consequences such as increased drug use and decreased treatment seeking.^17^ The Michigan study delineated several best practices for implementing such a program, including the importance of education to address stigma, reducing the administrative burden associated with distributing kits to frontline providers, studying the effect of leave-behind naloxone on 911 calls, and clarifying legal repercussions to mitigate fear of legal action against providers. Pennsylvania’s Senate Bill 95 of 2025, recently signed into law, provides an example of legislation that explicitly permits EMS providers to leave-behind naloxone.^18^

An unexpected finding in our study was that most naloxone kits were distributed mostly to patients who declined transport (73%). This included patients who had not only an overdose reversed but also patients with other chief complaints. This finding may represent a missed opportunity to initiate opioid use disorder treatment or reflect evolving beliefs and practices among people who use drugs regarding engagement with health-care systems. By contrast, a 1-year evaluation of a leave-behind naloxone program in Vermont found that 31.6% of at-risk individuals declined transport.^19^ Although the mortality of patients who declined transport after overdose appears to be low,^20^ the 1-year mortality of individuals treated by EMS who initially survive is high (7.5% in 1 study).^8^ Transporting to the emergency department is an opportunity to start medication for opioid use disorder**,** provide additional health-care interventions, and create linkage to ongoing treatment.^21^ In successful pilot programs, first responders have also started buprenorphine, particularly to relieve withdrawal symptoms after reversal of an overdose with naloxone.^22^, ^23^, ^24^, ^25^

A unique component of our study was the geospatial analysis to identify hotspots for prestationing naloxone in public-access boxes. Previous work from Cambridge, Massachusetts, found that positioning naloxone at 3-cluster centroids could place 40% of overdose events within 200 meters of a kit.^26^ Our threshold of 3 kits within a 600-meter radius to define a hotspot has implications for agencies and organizations planning to prestation naloxone in their own communities. A modeling study of public-access naloxone sites in Vancouver, Canada, found that strategically placing naloxone kits at transit stops was the most effective location to increase access to naloxone at times and places where overdoses are most likely to occur.^27^ Our observation that nearly all distribution hotspots were adjacent to transit stops aligns with these studies and suggests that transit hubs may be ideal sites for prestationed naloxone, though with the operational caveats regarding temperature and inventory control, as discussed above. Best practices for designing a public-access naloxone program at transportation stations have been described.^28^

In conclusion, implementation of a county-wide, multiagency leave-behind naloxone program was feasible and resulted in the distribution of >2400 kits in its first year. Paramedics and the STAR community response mobile crisis units delivered most kits, with distribution by police and fire less frequent. Geospatial analysis identified clusters near transportation hubs, suggesting that transit-focused prestationing could enhance access to naloxone at the time and location where overdose-related 911 calls tended to occur. High patient declination of transport indicates missed opportunities for engaging patients with opioid use disorder in further treatment, such as linkage to care and initiating medication for opioid use disorder. These results support multiagency collaboration and geospatial targeting to expand naloxone access. Further research should evaluate kit utilization, impacts on overdose outcomes, and strategies to improve linkage to treatment, especially given the high 1-year mortality after nonfatal overdoses.

## Author Contributions

JJ, DS, and SGW conceived and designed the study. JJ, AM, and KP supervised the conduct of data collection. JJ managed the data. JJ and SGW analyzed the data. JJ and SGW drafted the manuscript, and all authors contributed substantially to its revision. SGW takes responsibility for the paper as a whole.

## Funding and Support

This work was funded by The Naloxone Project, a nonprofit organization. Dr. Weiner’s effort was provided by NIH Grant 5R01DA05831. The views and conclusions contained in this document are those of the authors and should not be interpreted as representing the official policies or stance, either expressed or implied by the funding organizations.

## Conflict of Interest

The authors report no conflicts of interest associated with this manuscript. Outside of this work, Dr Weiner reports financial support from the 10.13039/100000026National Institute on Drug Abuse and consulting or advisory relationships with Vertex Pharmaceuticals, Inc, and Cessation Therapeutics, Inc. The other authors declare that they have no known competing financial interests or personal relationships that could have appeared to influence the work reported in this paper.
