# Supplementary material for: Assessment of a County-Wide, Pan-First Responder Take-Home Naloxone Program in Denver, Colorado
Source: J Am Coll Emerg Physicians Open. 2026 Mar 13;7(3):100357. doi: 10.1016/j.acepjo.2026.100357 (PMC12990331; doi:10.1016/j.acepjo.2026.100357)
Supplement: Supplementary Appendix [file mmc1.pdf]

Remove Welcome Page

TEST CPACC Naloxone Dispensing Form

Please complete this short form on-scene!

Click to add question count

START

Agency *

Type a description

Please Select

Address of Distribution *

Simply click the blue map pin icon to auto-populate, or type in your location. Please do NOT use any unit or suite numbers.

Street Address

Street Address Line 2

City

Search

State

Zip Code

Did the individual at risk of overdose receive an administered dose of naloxone prior to your arrival? *

Type a description

- ◯ This call did not involve an overdose.
- ◯ No, patient did NOT receive naloxone prior to our arrival.
- ◯ Yes, naloxone was administered by a COMMUNITY MEMBER prior to our arrival.
- ◯ Yes, naloxone was administered by a FIRST RESPONDER prior to our arrival.

How many naloxone kits did you distribute to the individual at risk of overdose? *

Type a description

1

How many naloxone kits did you distribute to individuals in a position to assist those at risk for an opioid overdose? *

Type a description

0

Was the indvidual at risk for overdose transported to the hospital? *

Type a description

- ◯ Yes
- ◯ NA, community outreach interaction
- ◯ No, individual was not transported to the hospital
- ◯ No, individual was a standing order refusal
- ◯ No, the individual refused against medical advice
- ◯ Unknown

OPTIONAL: Please share any additional (non-sensitive or identifiable) details about your distribution of naloxone.

What went well, or what didn't work well? What context would help? Don't share sensitive information.

SUBMIT

HIPAA
COMPLIANCE

Edit Thank You Page
